# ESCRT-Independent Budding of HIV-1 Gag Virus-Like Particles from *Saccharomyces cerevisiae* Spheroplasts

**DOI:** 10.1371/journal.pone.0052603

**Published:** 2012-12-21

**Authors:** Andrew P. Norgan, Jacqueline R. E. Lee, Andrea J. Oestreich, Johanna A. Payne, Eugene W. Krueger, David J. Katzmann

**Affiliations:** 1 Department of Biochemistry and Molecular Biology, Mayo Clinic, Rochester, Minnesota, United States of America; 2 Center for Cell Signaling in Gastroenterology, Mayo Clinic, Rochester, Minnesota, United States of America; Boston College, United States of America

## Abstract

Heterologous expression of HIV-1 Gag in a variety of host cells results in its packaging into virus-like particles (VLPs) that are subsequently released into the extracellular milieu. This phenomenon represents a useful tool for probing cellular factors required for viral budding and has contributed to the discovery of roles for ubiquitin ligases and the endosomal sorting complexes required for transport (ESCRTs) in viral budding. These factors are highly conserved throughout eukaryotes and have been studied extensively in the yeast *Saccharomyces cerevisiae*, a model eukaryote previously utilized as a host for the production of VLPs. We used heterologous expression of HIV Gag in yeast spheroplasts to examine the role of ESCRTs and associated factors (Rsp5, a HECT ubiquitin ligase of the Nedd4 family; Bro1, a homolog of Alix; and Vps4, the AAA-ATPase required for ESCRT function in all contexts/organisms investigated) in the generation of VLPs. Our data reveal: 1) characterized Gag-ESCRT interaction motifs (late domains) are not required for VLP budding, 2) loss of function alleles of the essential HECT ubiquitin ligase Rsp5 do not display defects in VLP formation, and 3) ESCRT function is not required for VLP formation from spheroplasts. These results suggest that the egress of HIV Gag from yeast cells is distinct from the most commonly described mode of exit from mammalian cells, instead mimicking ESCRT-independent VLP formation observed in a subset of mammalian cells. As such, budding of Gag from yeast cells appears to represent ESCRT-independent budding relevant to viral replication in at least some situations. Thus the myriad of genetic and biochemical tools available in the yeast system may be of utility in the study of this aspect of viral budding.

## Introduction

*Saccharomyces cerevisiae* is a model for probing fundamental eukaryotic cellular processes, including the ability to serve as a host during viral replication and release. Multiple observations suggest that yeast may serve as a suitable model system in which to study a wide variety of viral processes. Early work establishing yeast as a model for studying viral life cycles included the demonstration of internalization and intracellular trafficking of Rous sarcoma virus (RSV) and Semliki Forest virus in yeast cells lacking their cell wall (spheroplasts), and the observation that expression of Hepatitis B virus surface antigen leads to the assembly of particles similar to those formed in human cells [1], [2]. Later investigations with Brome mosaic virus showed that yeast can be stably transformed with RNA episomes, indicating that the cellular factors required for viral RNA transcription and replication are present [3]. The first non-endogenous infectious virus produced in yeast was reported by the Ahlquist group, which observed the *de novo* synthesis of infectious Flock house virus from RNA transfected *S. cerevisiae* [4]. These observations demonstrate that yeast can be a useful model for the study of aspects of viral life cycles.

Among viruses the components and host factors necessary for replication vary, yet generalities in mechanism exist. For many enveloped viruses, including the retroviruses (e.g., HIV, Human T-cell Lymophotropic virus, Equine Infectious Anemia virus, RSV), Rhabdoviruses (e.g., Vesicular Stomatitis virus (VSV) and Rabies virus), Filoviruses (e.g., Ebola virus and Marburg virus), and Arenaviruses (e.g., Lymphocytic choriomeningitis virus and Lassa virus), a common process encompasses the final step of replication, viral budding [5]–[14]. A shared characteristic of the aforementioned viruses is the possession of one or more short amino acid sequences called late (L-) domains, named for the late stage budding defect observed upon their mutation or deletion [15]–[17]. The three canonical L-domains, P(T/S)AP, 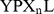 and PPXY, facilitate viral interactions with the cellular endosomal sorting complexes required for transport (ESCRT) proteins by binding either mammalian ESCRT-I protein TSG101 (yeast homolog Vps23), ESCRT-associated protein ALIX (yeast homolog Bro1), or the WW domains of Nedd4 (yeast homolog Rsp5) family ubiquitin ligases, respectively (reviewed in [18]). Although L-domains interact with different cellular factors, they are sometimes interchangeable, and may also function synthetically to promote efficient viral budding [19], [20]. The ESCRTs, a series of four multi-protein complexes ESCRT-0, -I, -II, -III, and associated proteins Vps4 and ALIX/Bro1, were first described in yeast (reviewed in [21]–[24]). Their endogenous cellular functions are to facilitate the trafficking of ubiquitin modified intracellular membrane-bound proteins to the lysosome for degradation, and to assist in cellular abscission and autophagy (reviewed in [25], [26]). The specific function of the ESCRT proteins in viral budding is not yet fully defined, but appears to be related to the ability of ESCRTs to drive topologically-relevant membrane deformation and scission [27] (reviewed in [28], [29]).

The use of yeast as a model system in which to study the interactions and mechanisms involved in viral replication and budding has been an appealing concept, as the ESCRT proteins and associated factors are highly conserved from yeast to humans and much success has been found studying ESCRT functions in yeast [21], [23]. Indeed, yeast have been successfully used as a surrogate host cell in which to study Tomato bushy stunt virus (tombusvirus) interactions with the Nedd4-type ligase Rsp5, ESCRT protein Vps23, and other cellular proteins required for its replication [30], [31]. Viral budding studies in yeast are made practical by the ability of many enveloped virus structural proteins, independently of nucleic acid or viral non-structural proteins, to drive their own membrane localization, assembly, and budding [32]. The HIV protein pr55 Gag, for example, drives the formation and budding of ‘viral-like particles’ (VLPs) when expressed in mammalian or insect cells [33]–[36]. When expressed in yeast, Gag is similarly myristoylated, trafficked to the plasma membrane and incorporated into VLPs [37], [38]. However, HIV Gag VLPs (or VLPs derived from RSV) are not released from the yeast cell as a consequence of interference by the cell wall [39], [40]. The arrest of VLP budding can be overcome by enzymatic removal of the cell wall, as has been demonstrated by the budding of HIV Gag VLPs from yeast spheroplasts [41].

Yeast-derived Gag-containing VLPs form fully lipid-enclosed spherical particles of variable electron density, with a buoyant density slightly less than that observed with Gag particles produced from Sf9 insect cells [41]. As with Gag VLPs formed in mammalian cells, yeast-derived Gag VLPs require the C-terminal Capsid domain of Gag for proper assembly and particle formation [41]. Interestingly, and in contrast to reports from experiments performed in certain cell lines, Gag VLPs derived from yeast do not appear to require the p6 domain (which encompasses the Gag L-domains) for budding [5], [41]–[43]. Additional experiments have shown that a Gag chimera in which firefly luciferase protein has been appended to the carboxyl-terminus buds efficiently from yeast; a finding consistent with experiments in mammalian cell lines in which epitope-tagged forms of Gag fused with the V3 loop of HIV-1 envelope protein or green fluorescent protein (GFP) are competent for budding independent of wild-type protein [44]. In addition, HIV Gag VLPs produced in yeast stimulate CD4+ and CD8+ T-cells [45]. Thus, many of the characteristics of HIV Gag VLPs produced in human cell lines are present in yeast-derived VLPs. Therefore, we hypothesized that the yeast system, with its robust genetics and highly conserved complement of ESCRT and ESCRT-associated proteins, might serve as an effective tool for probing host and viral factors necessary to facilitate HIV Gag budding. In this study, we observed: 1) HIV Gag L-domains are not required for incorporation of Gag-GFP into VLPs in yeast; 2) incorporation of Gag-GFP into VLPs occurs in the absence of ESCRT function; 3) incorporation of Gag-GFP into VLPs is not impacted by loss of function alleles of the essential HECT ubiquitin ligase Rsp5. These results are consistent with observations of ESCRT-independent viral budding in some mammalian cells lines, and raise questions about how, precisely, membrane deformation and scission for HIV budding are accomplished in the absence of functional ESCRT proteins.

## Results

### Expression and Localization of HIV Gag-GFP Fusion Proteins

**Figure 1:**
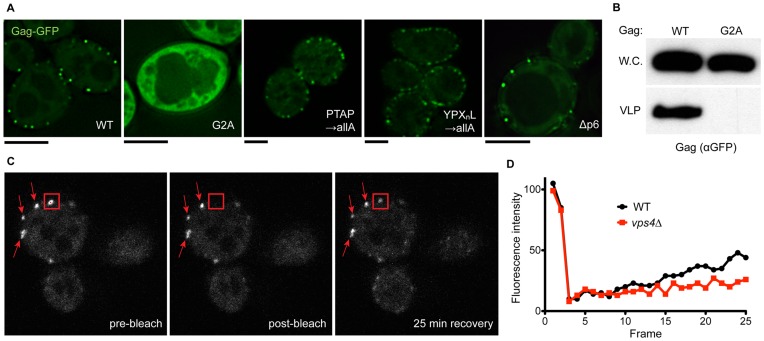
Expression and localization of HIV Gag-GFP fusion proteins. (A) Live cells (SEY6210 or JPY69 for 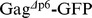) expressing the indicated form of Gag-GFP were visualized by fluorescence microscopy. Scale bar for each image represents 4 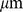. (B) Yeast cells expressing either WT Gag-GFP or 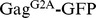 were analyzed for their ability to incorporate these chimeras into VLPs. Whole cell extracts (W.C.) and the VLP fractions were subjected to SDS-PAGE and western blotting with anti-GFP antibody to allow visualization. (C) Representative images of fluorescence recovery after photobleaching performed on wild type cells expressing Gag-GFP (pre-bleach, first image captured post-bleach, and 25 minute recovery). The red box highlights a punctum that was subjected to bleaching while the red arrows highlight Gag-GFP puncta that were not. (D) Quantification of fluorescence recovery after photobleaching (at one frame per minute) for wild type and 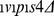 cells expressing wild type Gag-GFP.

HIV Gag-GFP fusion proteins were expressed in *S. cerevisiae* and their subcellular localization was assessed by fluorescence microscopy of live cells. Wild type Gag-GFP displayed a punctate plasma membrane distribution, while the myristoylation mutant 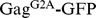 mutant showed cytosolic localization (Fig. 1A). Equivalent expression of the Gag-GFP fusion proteins was verified by Western blotting of whole cell lysates made from cells expressing Gag-GFP or 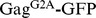 (Fig. 1B, upper panel). The dynamic behavior of these Gag-GFP structures was observed using time-lapse analyses of live single cells expressing wild type Gag-GFP, and revealed no visible movement over a 25 minute course (for example, see structures highlighted with arrowheads in Figure 1C). The stability of these structures allowed for analysis of dynamic exchange of Gag-GFP therein. Fluorescence recovery after photobleaching (FRAP) revealed that there is dynamic exchange of Gag-GFP at these stable, punctate structures; approximately 45% of the signal was recovered over a 25 minute time course (for example see boxed region in Figure 1C, quantitation in Figure 1D). Additionally, fluorescence loss in photobleaching (FLIP) was performed in order to determine whether the cytosolic pool of Gag-GFP was contributing to the recovery of Gag-GFP puncta at the cell periphery. Over a 30 minute time course we observed an 85% drop in the cytosolic fluorescence of cells undergoing cytosolic bleaching (by contrast only 4% drop in neighboring cells not undergoing bleaching) and concomitant 60% drop in the signal at peripheral Gag-GFP puncta (data not shown). Together, these data reveal that the punctate fluorescence pattern observed at the plasma membrane of cells is undergoing dynamic exchange with the cytosolic pool of Gag-GFP.

In multiple, but not all, mammalian cell types it has been shown that the C-terminal HIV Gag p6 L-domains comprised of amino acid sequences 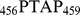 and 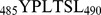 contribute to efficient HIV budding by facilitating interaction with host cell proteins (reviewed in [46]). To examine the impact of the HIV Gag L-domains on Gag-GFP subcellular localization, constructs were generated with the Gag L-domain motifs 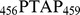 or 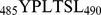 mutated to alanine residues (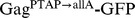; 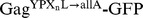), or the p6 domain deleted (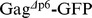). Mutation of the PTAP or YPLTSL sequences or deletion of the p6 domain displayed no apparent subcellular localization defect (Figure 1A). Together, these data reveal that Gag-GFP forms stable structures that undergo some level of dynamic exchange. As the only mutant defective for this morphology was the myristoylation-defective form, it would appear that delivery of Gag-GFP to these structures is dependent upon this lipid modification and not motifs responsible for associating with cellular machinery involved in L-domain-dependent budding.

### Release of HIV Gag-GFP VLPs from *S. cerevisiae* Spheroplasts

Previous studies have demonstrated that expression of HIV Gag in yeast spheroplasts results in packaging of Gag into viral-like particles (VLPs). To examine the ability of Gag-GFP to incorporate into VLPs, cells expressing the Gag-GFP fusion protein were grown in minimal media, removed of their cell wall (spheroplasted), and incubated in media with osmotic support to allow release of VLPs. The putative VLP fraction was collected based on previous protocols and Gag-GFP was detected in whole cell extract or VLP fraction by western blotting with anti-GFP antibody. WT Gag-GFP, but not the myristoylation-defective protein (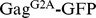), was detected in the extracellular supernatant fraction putatively containing VLPs (Fig. 1B). Together, these data suggest that the appearance of Gag-GFP in the extracellular fraction is dependent upon myristoyl modification and localization to punctate structures within the plasma membrane.

**Figure 2:**
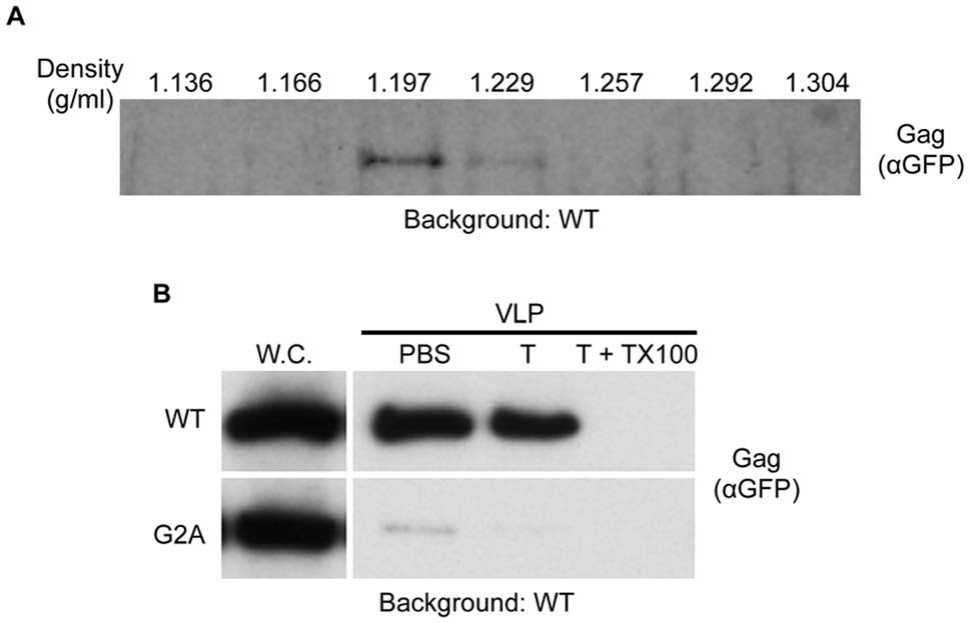
Characterization of VLPs. (A) VLPs were collected from wild type cells expressing wild type Gag-GFP and subjected to centrifugation across a sucrose gradient (20–70%, weight to volume). 1 ml fractions were subsequently collected, the density of each fraction was measured, and each fraction was subjected to SDS-PAGE and anti-GFP immunoblotting. (B) Wild type cells expressing either Gag-GFP (WT) or 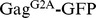 (G2A) were equivalently processed to isolate the whole cell and VLP fractions. The VLP fraction was subsequently incubated with either PBS alone (PBS), PBS+Trypsin (T), or PBS+Trypsin+Triton X-100 (T+TX100), and post reaction material was visualized by immunoblotting using anti-GFP antibody. Whole cell lysates (W.C.) from cells expressing either WT Gag-GFP or 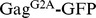 used to generate the VLP fraction were loaded onto the gel to indicate the relative amount of expression.

Further characterization of this extracellular fraction was performed to confirm Gag-GFP incorporation into VLPs. First, membrane association of extracellular Gag-GFP was addressed by analyzing the buoyant density of this material. Equilibrium centrifugation of the putative VLP fraction over a 20–70% (wt/vol) sucrose gradient followed by western blotting of resulting fractions with anti-GFP antibody revealed a buoyant density of approximately 1.2 g/ml (Figure 2A). A previous report indicated that VLPs generated from yeast expressing Gag (without a GFP tag) display comparable buoyant density, consistent with the idea that Gag-GFP is membrane associated [41]. Second, to establish that the Gag-GFP-containing extracellular membranes collected represented fully lipid-enclosed particles, and not merely membranes of the appropriate density with associated Gag-GFP, the putative VLP fraction from cells expressing Gag-GFP or 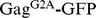 was processed identically and subsequently treated to protease treatment with or without the detergent Triton X-100. Gag-GFP present in the VLP fraction was protected from trypsin-mediated proteolysis in the absence of detergent yet was degraded in the presence of trypsin and Triton X-100 (Figure 2B). By contrast, the 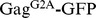 sample displayed only a small amount of immunoreactive material in the VLP fraction, and this was susceptible to trypsin-mediated degradation even in the absence of detergent (Figure 2B). These data support the interpretation that while the VLP fraction has a low-level of contamination from soluble proteins, the majority of Gag-GFP present in this fraction is contained within lipid vesicles of a buoyant density consistent with previously described yeast VLPs [41]. Together, these data highlight the connection between appearance of Gag-GFP in punctate plasma membrane regions and the presence of Gag-GFP in the extracellular fraction in a lipid-enclosed particle (aka VLP). Additionally, a C-terminal GFP tag does not disrupt HIV Gag expression, localization, or budding of lipid enclosed viral-like particles in yeast.

**Figure 3:**
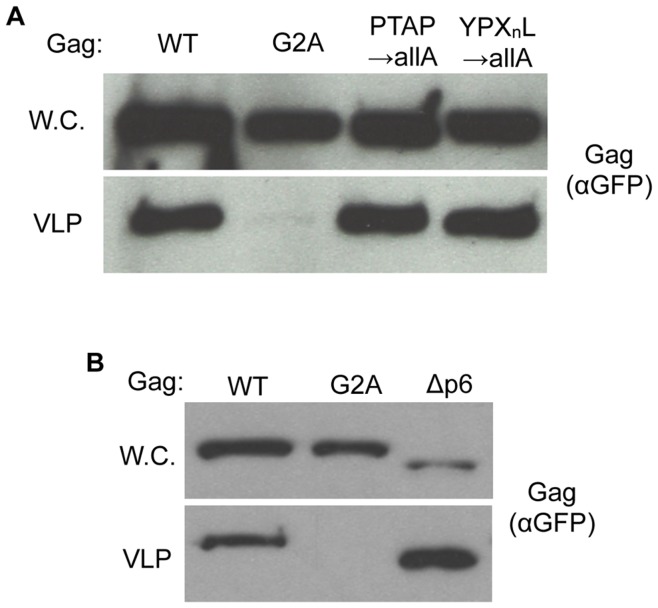
HIV Gag-GFP VLP budding from yeast spheroplasts does not require Gag L-domains. (A) and (B) Wild type yeast were transformed with 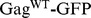, 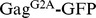, 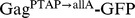, 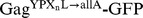 or 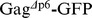. VLP budding of the Gag-GFP constructs was assayed as described in the Methods section. Gag-GFP was visualized by Western blotting of whole cell extracts and VLP fractions with anti-GFP antibody.

To examine the impact of the HIV Gag L-domains on Gag-GFP budding, VLP formation assays were conducted with the 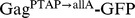, 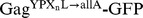, and 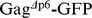 constructs. Mutation of either the PTAP or YPLTSL sequences displayed no apparent defect in either subcellular localization or VLP production efficiency (Figures 1A and 3A). Similarly, deletion of the entire p6 domain showed no alteration in subcellular localization or reduction in VLP budding (Figures 1A and 3B). These data are consistent with previous experiments demonstrating no defect in VLP production from spheroplasts expressing Gag containing a p6 domain deletion [41]. These findings suggest that yeast may represent a suitable model for probing L-domain-independent budding from host cells.

### Activity of the Nedd4 Homolog Rsp5 is not Responsible for Bypass of L-domain Requirement

**Figure 4:**
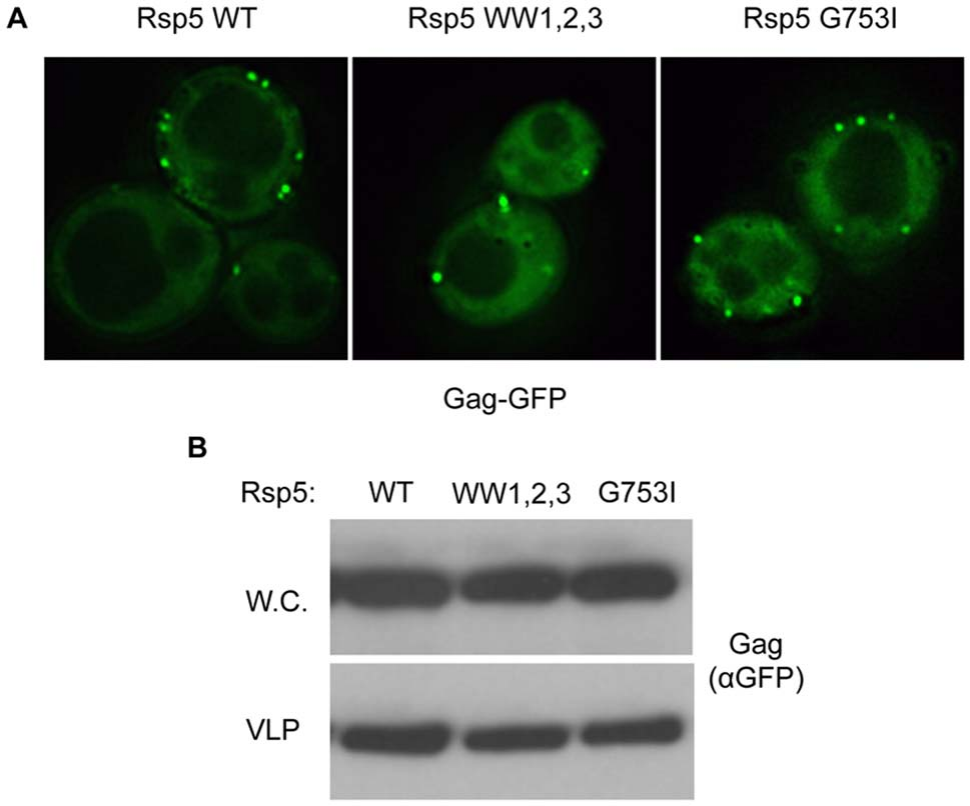
HIV Gag-GFP VLP budding from yeast spheroplasts is not diminished by loss-of-function mutations in Rsp5. 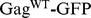
 was transformed into an 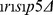 background complemented with 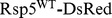, 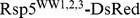, or 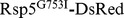. (A) Localization of 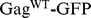 by fluorescence microscopy on the indicated live cells. (B) VLP budding assays were conducted with cells of the indicated genetic backgrounds, and whole cell extracts (W.C.) and VLP fractions were visualized by anti-GFP immunoblotting.

The ability of L-domain-mutated forms of Gag to enter yeast VLPs lead us to investigate potential contributions to VLP budding from the HECT ubiquitin ligase and Nedd4 homolog Rsp5, which has been demonstrated to impact multiple endomembrane sorting events [47]–[49]. Nedd4 family ubiquitin ligases have been shown to rescue the budding of L-domain-deficient structural proteins [50]–[53]. Rsp5 is the only Nedd4 family member in yeast; expression of Rsp5 in mammalian cells partially rescues L-domain-deficient viral structural proteins [53]. As Rsp5 is an essential gene, its role in L-domain-independent incorporation of Gag-GFP into yeast VLPs was analyzed using a loss of function alleles defective for either ubiquitin ligase activity (G753I) or protein-protein interaction via the WW domains (WW1,2,3 triple mutant) [54]. While cells expressing these mutant forms of Rsp5 have previously been demonstrated to display severe defects in endomembrane protein trafficking [54], no defects in either subcellular localization of Gag-GFP or incorporation of Gag-GFP into VLPs was observed (Figures 4A and 4B). These data indicate Rsp5 function is not required for the incorporation of Gag-GFP into VLPs. Taken together with previous data, this would indicate that Gag targeting to the plasma membrane and into VLPs does not require known interactions with cellular machinery (either L-domains or the L-domain bypass mechanism mediated by Nedd4 family members, of which Rsp5 is the only member in yeast).

### Incorporation of Gag-GFP into Yeast VLPs does not Require ESCRT Function

**Figure 5:**
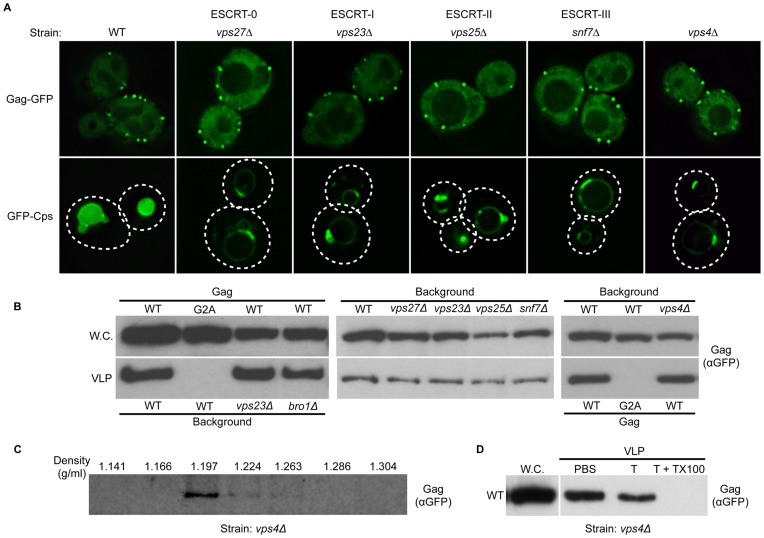
HIV Gag-GFP VLP budding from yeast spheroplasts is independent of ESCRT function. (A) 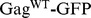 or GFP-Cps1 were transformed into wild type cells or strains deleted for a component of each ESCRT complex, ESCRT-0 (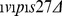), ESCRT-I (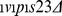), ESCRT-II (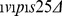), ESCRT-III (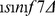) or VPS4 (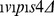), and GFP localization was assessed by fluorescence microscopy of live cells. In the wild type background GFP-Cps1 was sorted into the lumen of the yeast vacuole, while loss of ESCRT function, Bro1 function, or Vps4 function resulted in missorting to the vacuolar limiting membrane and accumulation within the aberrant class E compartment. (B) 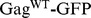 or 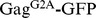 were expressed in the indicated strains strains and whole cell extracts (W.C.) and VLP fractions were analyzed by Western blotting with anti-GFP antibody. (C) VLPs were collected from 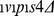 cells expressing wild type Gag-GFP and subjected to centrifugation across a sucrose gradient (20–70%, weight to volume). 1 ml fractions were subsequently collected, the density of each fraction was measured, and each fraction was subjected to SDS-PAGE and anti-GFP immunoblotting. (D) 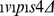 cells expressing wild type Gag-GFP were processed to isolate the whole cell and VLP fractions. The VLP fraction was subsequently subjected to incubation with either PBS alone (PBS), PBS+Trypsin (T), or PBS+Trypsin+Triton X-100 (T+TX100) and the reactions were visualized by immunoblotting using anti-GFP antibody. Whole cell lysate (W.C.) from the cells used to generate the VLP fraction was loaded to indicate expression.

Experiments in mammalian cells have identified components of the ESCRT proteins, including ESCRT-I (specifically TSG101), ESCRT-III, and the factors Vps4 and ALIX, that are required for maximal budding efficiency of HIV [18]. Although no dependence upon L-domains was observed with regard to Gag-GFP subcellular localization and incorporation into VLPs in the yeast system, the possibility remained that Gag budding in yeast would require the membrane deformation and scission activities of of ESCRT-III and associated factors during budding. To examine the requirements for ESCRT function in the context of yeast, the production of Gag-GFP containing VLPs was examined in yeast backgrounds deleted for components of each of the ESCRTs (-0, -I, -II, and III), the yeast ALIX homolog Bro1, or the AAA-ATPase Vps4 (which is essential for ESCRT function in all contexts examined to date). HIV 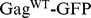 localization, expression and VLP production was assayed in the *S. cerevisiae* backgrounds 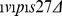 (ESCRT-0), 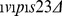 (ESCRT-I), 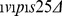 (ESCRT-II), 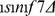 (ESCRT-III), 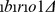 and 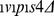 [55]–[60]. These strains were transformed with either the previously characterized MVB cargo GFP-Cps1 or Gag-GFP, and subcellular localization of these chimeric proteins was documented by live cell fluorescence microscopy. Consistent with previous observations, the prototypic MVB cargo GFP-Cps1 was sorted into the lumen of the vacuole (the functional equivalent of the lysosome) via the MVB pathway in WT cells. Loss of ESCRT function, Bro1 function, or Vps4 function resulted in a defect in MVB sorting as evidenced by missorting of GFP-Cps1 to the limiting membrane of the vacuole (rather than the lumen) and accumulation within the aberrant class E compartment (Figure 5A). Although these mutant strains displayed clear defects in MVB sorting, they displayed no defect in subcellular localization of Gag-GFP at the plasma membrane, nor did they impact the production of Gag-GFP VLPs (Figures 5A, 5B, and data not shown). The dynamic nature of Gag-GFP punctate plasma membrane structures was addressed by FRAP in 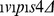 cells, and kinetics of recovery were comparable to wild type cells (Figure 1D). In order to address the possibility that the viral particles detected upon perturbation of ESCRT function were somehow aberrant or unlike VLPs produced in the wild type context, VLPs produced in the 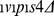 background were subjected to sucrose gradient fractionation and protease protection assays. The apparent density of Gag VLPs produced in 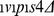 cells was approximately 1.2 g/ml, indistinguishable from particles formed in wild type cells (Figures 5C and 2A). As with VLPs produced in wild type cells, VLPs from the 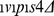 strain were protected from trypsin-mediated degradation in the absence of detergent (Figure 5D). These data indicate that incorporation of Gag-GFP into VLPs and formation of these vesicles is not dependent upon ESCRT function.

## Discussion

Previous studies have documented the use of yeast as a host cell capable of generating Gag-containing VLPs, however those studies also highlighted the lack of dependence upon the L-domains [41]. Our findings are entirely consistent with previous results as Gag-GFP was present in the VLP fraction regardless of mutation of the PTAP or YPXL L-domains, or even deletion of the p6 domain. Work in mammalian cell systems has underscored the ability of Nedd4 family members to support the budding of L-domain-mutant forms of Gag [51], [53]. While the mechanism of late budding rescue by Nedd4-family ubiquitin ligases is not well understood, it appears to involve the ubiquitination of Gag with K63-linked ubiquitin chains that may provide an alternative surface for ESCRT protein engagement outside of L-domain motifs [53]. Mammals possess nine Nedd4 family members with high similarity and redundancy, complicating investigation of the interplay between Gag and Nedd4 family members [reviewed in [61]]. In contrast, Rsp5 is the only Nedd4 homolog in *S. cerevisiae*. We examined the possibility that L-domain-independent budding in yeast was due to the activity of the sole yeast Nedd4 homolog Rsp5.

In order to execute these studies, it was first necessary to confirm the ability of yeast cells to incorporate Gag (in this case Gag-GFP) into VLPs (Figure 2) independent of L-domain interaction surfaces (Figure 3). As *RSP5* is an essential gene, previously characterized loss-of-function alleles defective for either protein-protein interaction via the WW domains (WW1, 2, 3) or ubiquitin ligase activity (G753I) were subsequently utilized. While these alleles have previously been characterized as displaying defects in endomembrane trafficking [54], [62], they displayed no alterations in either the subcellular localization of Gag-GFP or its ability to enter VLPs (Figure 4 and data not shown). This is consistent with the interpretation that Rsp5 function is not required for the formation of Gag-GFP-containing VLPs and as such is not the mechanism responsible for L-domain-independent incorporation of Gag into yeast ILVs. However, the experiments utilized hypomorphic alleles due to the essential nature of Rsp5 and as such it is possible that residual activity of the encoded proteins, while insufficient for known Rsp5 functions such as endocytic trafficking, is sufficient to support the generation of Gag-GFP-containing VLPs. Additional studies pertaining to the mode of interaction between Nedd4 family members and Gag are required to resolve this issue definitively, but no role for Rsp5 in incorporation of Gag-GFP into yeast VLPs was identified in the present study.

Efficient budding of HIV-1 and a number of enveloped viruses from mammalian cells has been demonstrated to depend on the function of at least a subset of ESCRT proteins [63], [64]. At first glance this would appear to contrast our present findings in which no defect in sorting of Gag-GFP into VLPs was observed. Similar findings have recently been generated by another laboratory (Y. Morikawa, personal communication). One possible explanation would be that Gag-GFP is exiting yeast cells via unconventional secretion, however this seems unlikely by virtue of unconventional secretion’s ESCRT-dependence [65]. Another possibility stems from previous studies documenting ESCRT-independent viral budding. In the case of VSV mutation of the M protein PPXY motif significantly impairs viral budding but renders the virus insensitive to inhibition by dominant negative VPS4 [66]. Similarly, mutation of the three L-domains in Moloney murine leukemia virus (MoMLV) reduces budding to approximately 15% of WT levels, but also renders MoMLV insensitive to inhibition by dominant negative VPS4 [67]. The simplest interpretation of these data is that a subset of VLPs do not require ESCRT function, but utilize instead a secondary egress mechanism. Whether such a mechanism relies on hijacking a cellular process (e.g., exosomes) or is instead mediated purely by viral proteins remains to be investigated.

Of particular relevance to the present work, it has also been reported that HIV-1 Gag lacking a functional L-domain buds at 20% or greater compared to wild type Gag in Jurkat T-cell or primary monocyte derived macrophage lines, although HeLa cells display a greater dependence upon L-domains [68], [69]. Reduced levels of L-domain-defective Gag incorporation into VLPs can be restored to wild type levels by the replacement of the HIV NC-p1-p6 with a leucine zipper in 293T cells, even in the presence of dominant negative CHMP3 (a mutant form of the ESCRT-III subunit that is a potent inhibitor of wild type Gag budding) [70]. This ESCRT-independent budding does not appear to be a gain-of-function resulting from its chimeric nature, but rather appears the result of NC-p1 region of Gag [70]. This region contains an inhibitory element between the Gag NC and p6 domains (SP2 or p1) [71]. Mutation of Gag SP2 results in near wild type levels of budding in the absence of the Gag PTAP motif, or the entire p6 domain [71]. These results are potentially explained by the existence of alternative modes of Gag-ESCRT interaction, such as the interaction between the Gag NC and Bro1 [72], [73]. Alternatively, these results are consistent with the interpretation that ESCRT function is not a requisite for the generation of Gag-containing VLPs. This appears to be the case in the present work, as deletion of Vps4 would be expected to produce dysfunction in any ESCRT-dependent process. One possibility consistent with both previous reports and our present findings is that HIV Gag budding may not intrinsically require ESCRT function, but rather that ESCRTs are needed to assist the virus in overcoming some form of cellular restriction of budding. In the absence of cellular restriction, for instance in yeast cells, Gag budding proceeds without ESCRT function. It is tempting to speculate that HIV budding in yeast might utilize a more primitive or basic budding mechanism that predates viral adaptation to, and restriction by, mammalian cells. For instance, one cellular phenomenon that could be usurped for viral budding is the the formation of exosomes/microvesicles. Exosomes have been proposed as a mode of HIV egress from cells (the ‘Trojan exosome’ hypothesis), and it has recently been demonstrated membrane anchored proteins are targeted to exosomes by default [69], [74]. Another possibility is that different lipid compositions of yeast and mammalian cell membranes may place distinct requirements on ESCRT function during the formation of extracellular vesicles. While the yeast system does not appear to represent a useful model for studying the contributions of ESCRT proteins to HIV budding, the system may be useful for studying other aspects of viral life cycle or industrial production of VLPs for the purpose of vaccine development efforts.

## Materials and Methods

### Strains

The following yeast strains were used in this study: SEY6210 (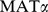 *leu2-3112 ura3-56* 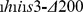 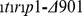 *lys2-801* 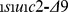; [75]), MBY21 (SEY6210; 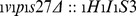; [76], EEY6-2 (SEY6210; 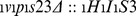; [56], BWY101 (SEY6210; 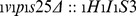; [57], EEY9 (SEY6210; 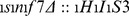; [58], MBY4 (SEY6210; 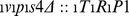; [60]) JPY69 (SEY6210; 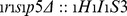, 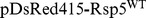; [54]), JPY74 (SEY6210, 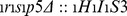, 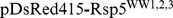; [54]), JPY89 (SEY6210, 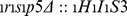, 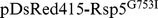; [54]).

### Plasmids


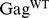
, 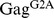, and 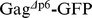 were generated by PCR amplification from an HIV-1 NL4-3 strain template with either EcoRI/NotI or BamHI/SalI restriction sites. pRS416-Gag-GFP was constructed by cloning Gag using EcoRI and NotI restriction sites in frame in the yeast expression plasmid pRS416 carrying the TDH3 promoter and a C-terminal GFP cassette. Mutants 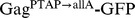 (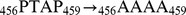) and 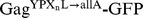 (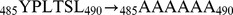) were made by site-directed mutagenesis using the GeneTailor (Invitrogen, Grand Island, NY) system. The construction of the pDsRed415-Rsp5 [54] and pGFP-Cps1 [77] plasmids has been previously described.

### Microscopy

Live cells were grown in minimal media for fluorescence microscopy. Micrographs were captured using a fluorescence microscope Olympus IX70 (Olympus, Center Valley, PA) with GFP and DsRed filters and a digital camera (Coolsnap HQ; Photometrics, Tucson, AZ). Deconvolution was performed with Delta Vision Softworx (Applied Precision, Issaquah, WA).

### Fluorescence Recovery after Photobleaching

The FRAP experiments were carried out on a Zeiss LSM510 confocal laser scanning microscope (Thornwood, NY). Transfected cells were observed with a 100×, 1.4 NA lens, and a region of interest was placed over a GFP labeled spot. A time lapse was started with 1 frame collected every 60 seconds. After the first 2 frames were collected, the region of interest was bleached for 50 iterations of the 488 nm laser at 100% laser power (approximately 1.5 seconds total bleach time), and then frames were collected every 60 seconds for the next 30 minutes. The collected images were subsequently aligned in Adobe Photoshop (San Jose, CA), and fluorescence intensities were determined using iVision software (BioVision, Exton, PA) on an Apple Macintosh computer (Cupertino, CA), and graphs were plotted using Microsoft Excel (Redmond, WA).

### VLP Budding Assay

Yeast transformants were grown at 30°C in defined minimal media (Yeast Nitrogen base plus glucose) to OD600 = 1.0–2.0. Spheroplasting was carried out as detailed in Sakuragi *et al.* with the following modifications: Spheroplasts were incubated in osmotically supportive rich media (Yeast-Peptone-Dextrose with 1 M sorbitol) for 2 hours at 30°C with gentle agitation [41]. Culture media was clarified by centrifugation in an Allegra 22-R at 4,5000 RPM for 20 minutes. Clarified supernatant was then centrifuged through a 30% (wt/vol) sucrose cushion at 4°C, 30,000 rpm for 2 hours. For VLP assays, VLP pellets were resuspended in 100 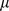
l 1X SDS Sample Buffer, heated at 100°C for 5 minutes, and visualized by Western blotting using monoclonal anti-GFP AV JL-8 (BD Bioscience, Franklin Lakes, NJ). For protease protection assays, VLPs were resuspended in 100 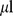 PBS, PBS with 1 mg/ml trypsin, or PBS with 1 mg/ml trypsin and 0.5% Triton X-100 and incubated at 37°C for 20 minutes and then combined with 50 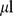 3X SDS Sample Buffer, heated at 100°C for 5 minutes, and visualized by Western blotting. For sucrose gradient fractionation, VLP pellets were resuspended with PBS, loaded onto 20%–70% (wt/vol) sucrose gradients and centrifuged at 4°C, 40,000 rpm for 12–18 hours. Gradients were fractionated and fraction densities measured by refractometry. Fractions were TCA precipitated with 2 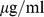 bovine serum albumin (BSA) carrier, and the precipitated material was sonicated and subjected to SDS-PAGE and Western blotting.
