# Correction: ESCRT-Independent Budding of HIV-1 Gag Virus-Like Particles from *Saccharomyces cerevisiae* Spheroplasts

**DOI:** 10.1371/annotation/bb8f73cc-4355-4198-b020-e9cdfb6ced86

**Published:** 2013-11-04

**Authors:** Andrew P. Norgan, Jacqueline R. E. Lee, Andrea J. Oestreich, Johanna A. Payne, Eugene W. Krueger, David J. Katzmann

Due to errors introduced during the production process, some of the text has been reproduced incorrectly.

In the Results section, in the sub-section "Incorporation of Gag-GFP into Yeast VLPs does not Require ESCRT Function", errors were introduced into the text. The correct text can be viewed here: http://www.plosone.org/corrections/pone.0052603.001.cn.tif 

In the Materials and Methods section, in the sub-section "Strains", errors were introduced into the text. The correct text can be viewed here: http://www.plosone.org/corrections/pone.0052603.002.cn.tif 

In the legends of Figure 1, Figure 4, and Figure 5, errors were introduced into the text. The correct legends can be found below.

Figure 1: http://www.plosone.org/corrections/pone.0052603.003.cn.tif 

Figure 4: http://www.plosone.org/corrections/pone.0052603.004.cn.tif 

Figure 5: http://www.plosone.org/corrections/pone.0052603.005.cn.tif 

